# Marine Macroalgae in Rabbit Nutrition—A Valuable Feed in Sustainable Farming

**DOI:** 10.3390/ani12182346

**Published:** 2022-09-08

**Authors:** Sabela Al-Soufi, Javier García, Antonio Muíños, Marta López-Alonso

**Affiliations:** 1 Departmento de Patoloxía Animal, Universidade de Santiago de Compostela, 27002 Lugo, Spain; 2 Departamento de Producción Agraria, Escuela Técnica Superior de Ingeniería Agronómica, Agroalimentaria y de Biosistemas, Universidad Politécnica de Madrid, C/Senda del Rey 18, 28040 Madrid, Spain; 3 Porto Muiños S.L., 15185 Cerceda, Spain

**Keywords:** macroalgae, seaweed, rabbit nutrition, alternative to antibiotics

## Abstract

**Simple Summary:**

Commercial rabbit farming has faced critical challenges in the last few years, during which the ban on the prophylactic use of antibiotics in animal feed has added to the weakness of the production system and a decrease in consumption of rabbit meat. Considering the potential role of macroalgae as an alternative to the use of antibiotics in animal nutrition, this review paper aims to evaluate the use of macroalgae in rabbit farming. It specifically focuses on how macroalgae can be used sustainably to improve rabbit health as an economically viable alternative that could help guarantee the future of this high-value sector.

**Abstract:**

The rabbit meat industry has faced critical challenges in the last few years, during which the ban on the prophylactic use of antibiotics in animal feed has added to the weakness of the production system and a decrease in consumption of rabbit meat. This review paper highlights the potential value of macroalgae in the rabbit farming sector as an alternative to the use of antibiotics to improve rabbit health. In line with sustainable agriculture programmes, the use of seaweed in rabbit nutrition may improve gut health according to the One Health approach, whereby consumers and the environment could receive tangible benefits. The inclusion of algae in animal feed has experimentally proven to help to reduce intestinal dysbiosis. However, further studies evaluating the prebiotic effects of algal components on gut health and also identifying the compounds directly responsible for the antimicrobial, antiviral, antioxidative and anti-inflammatory properties of algae are still needed. Furthermore, the inclusion of marine algae in rabbit food could potentially become a commercial marketing strategy that could attract new consumers who are concerned about environmental sustainability and who are looking for different, high-quality foods.

## 1. Introduction

Rabbit meat is less common than other types of meat (such as chicken, beef and pork) but is a valuable product in Mediterranean countries, where it was consumed by ancient civilizations and is still consumed in traditional gastronomy today [1]. The EU is the second world producer of rabbit meat (after China), with most production being concentrated in Spain, France and Italy [2]. Intensive rabbit farming became popular in these countries in the 1980s and led to a highly specialized industry, although there remain small farms still in need of modernization [2,3].

**Figure 1:**
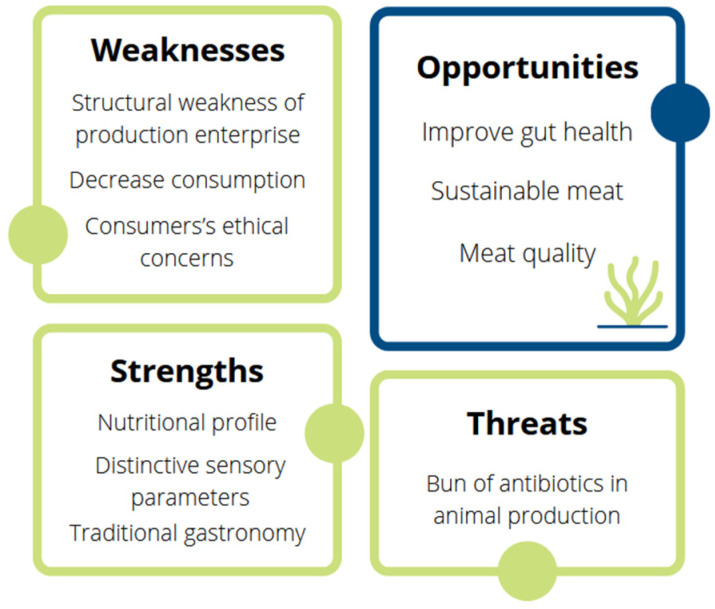
SWOT analysis of rabbit farming.

The rabbit farming industry is facing a critical, complex and challenging period characterised by structural weaknesses in the production systems and a gradual reduction in the consumption of meat (Figure 1) [2]. Within the farming systems, the main challenge is possibly to comply with the EU ban on the prophylactic use of antibiotics in animal production (Regulation (EC) No. 1831/2003). Similar to pig farming, rabbit production is highly dependent on the use of medicated feed to control the high mortality rates caused by Epizootic Rabbit Enteropathy (ERE), which is characterized by intestinal dysbiosis that severely affects rabbits in the post-weaning period [4,5]. In 2006, the EU banned the use of antibiotics as prophylactics and growth promoters and limited their indiscriminate use, in order to fight the emergence of antibiotic resistance (Regulation (EC) No. 1831/2003). However, the lack of alternatives to antibiotic-medicated feed is currently causing huge losses in farms, making production almost economically unsustainable [6,7]. Moreover, rabbit meat is mostly marketed as the whole carcass (with the head), and there are almost no processed products available for other more modern types of consumption. This presentation conflicts with the increasing perception of rabbits as pets and with the lack of habitual consumption of this meat by the younger population. In addition, rabbit meat is more expensive than other white meat, because of the higher production costs [1,2]. Finally, as with other intensive systems, consumers are increasingly concerned about the welfare of caged animals and about the environmental impact of farming practices, which are highly dependent on external outputs [8].

Overall, the rabbit farming sector urgently needs a comprehensive plan to respond to all of these challenges and thus to search for alternative and economically sustainable production systems in accordance with the emerging welfare standards, incorporating dietary strategies that can improve the intestinal health of rabbits after weaning to prevent serious mortalities due to ERE.

Including marine macroalgae in the rabbit diet may be an appropriate strategy in response to the aforementioned problems. Although scarcely used in this sector, algae have been experimentally tested in the diets of other livestock species (particularly pigs after weaning). The inclusion of algae in feed has experimentally proven to help to reduce intestinal dysbiosis in pigs and could potentially be an alternative in rabbit farming. Furthermore, the inclusion of marine algae in rabbit food could potentially become a commercial marketing strategy that could attract new consumers who are concerned about environmental sustainability and who are looking for different, high-quality foods; in this sense, it has already been demonstrated that the inclusion of algae in animal feed improves the quality of meat [9,10,11,12].

This review study aimed to evaluate the potential use of macroalgae in rabbit farming, particularly focusing on how including macroalgae in the rabbit diet could be a sustainable means for improving the health of rabbits via economically viable practices that could help to guarantee the future of this high-value sector.

## 2. The Post-Weaning Period Is Critical in Rabbit Production

One of the critical stages in rabbit production is the post-weaning period, because of the numerous changes that animals undergo and the associated sensitivity at this point [13]. Weaning takes place usually around 35 days of age, but solid food intake increases gradually from day 15. During this period, important physiological changes occur due to the rapid change from a diet exclusively based on milk to the ingestion of large amounts of solid food and water. This transition is accompanied by several physiological adaptations of the digestive and immune system, which must respond to this new diet [14]. The digestive system transforms at many levels, i.e., gastric pH falls sharply from >5 to 1.6 around weaning; caecotrophy begins to develop at 3 weeks of age; the intestinal microbiota evolves continuously from the first days after birth [15]; therefore, fermentation starts in the caecum. The enzymatic system and thus the digestive capacity develop gradually, reaching optimal levels at 45–50 days of age. Microbial fermentative activity produces volatile fatty acids (VFAs), which contribute to changing the digestive conditions [14,16].

Gut microflora and gut mucosa, i.e., epithelial cells and gut-associated lymphoid tissues (GALTs), maintain a dynamic equilibrium that is particularly fragile in young rabbits. Microbiota stabilises after 30–40 days of age and is highly influenced by the diet [13]. The endogenous microbiota provides many benefits for the animal beyond the supply of a good profile of amino acids and VFAs. It acts as a barrier to pathogens by preventing their entry into epithelial cells, competing for resources and producing substances that maintain an unfavourable environment, such as VFAs and antimicrobial substances. It is also decisive for the development of digestive immunity, as it determines the primary antibody repertoire in GALTs at 4–8 weeks of age [13,17].

At this stage, rabbits are very susceptible to suffering from digestive dysbiosis, which is commonly caused by Epizootic Rabbit Enteropathy (ERE), a syndrome already described many decades ago [18]. More recently, ERE appeared in France in 1996 and rapidly spread to other regions of Europe [4,19], currently being one of the most important pathologies in rabbit farm systems [6,20]. It causes morbidity up to 90% and high mortality rates (30–80%), mainly affecting young rabbits in the fattening period, between 6–8 weeks of age [4,21,22]. Rabbits are infected horizontally via oral–faecal and oral–oral contact [22]. Affected animals reduce their feed and water intake and present with a distended abdomen and mild diarrhoea [4,5,21,22]. Necropsy shows caecal impaction and the distension of the stomach and small intestine, which are filled with liquid and gas. Histologically, there are signs of inflammation and congestion [22], which are not observed macroscopically in any organ [4,21]. To date, no single species has been identified as the sole cause of the disease, but in most cases, specific strains of *Clostridium perfringens* are implicated, while others might participate in the control of the negative effects of ERE, such as specific strains of *Bacteroides fragilis* and *Bacteroides dorei* [5]. Nowadays, the aetiology of this syndrome is still not completely defined, and the participation or co-infection of different bacterial species is not discarded [6].

The high mortality and reduction in animal performance associated with ERE cause major losses in rabbit farm systems [6,19,20,23]. The disease has been controlled in the last few decades via treatment with antibiotics, mostly in the form of medicated feed [5]. However, antibiotics affect not only the pathogens but also the whole intestinal flora, causing imbalance and dysbiosis [22,24]. Reducing the use of antibiotics to prevent the emergence of resistance has become a priority strategy in the EU [25]. Research aimed at finding alternatives to antibiotics in rabbit farming is scarce compared with research involving other major sectors such as pigs or poultry. Consequently, rabbit breeding faces a major challenge in finding ways for maintaining production without using antibiotics. It is, therefore, necessary to be aware of the dietary and management requirements of rabbits in the post-weaning period to ensure intestinal balance [14,26].

## 3. Key Point: To Improve Gut Health

The limitations of the use of antibiotics in livestock feed has forced the animal feed industry to search for nutritional strategies, such as the inclusion of bioactive substances in the diet to improve animal health. The improvement of gut health has been shown to have the greatest potential to reduce the negative economic impact of reducing the administration of antibiotics during the post-weaning period. Thus, feed must be designed that is better adapted to the digestive physiology of the recently weaned rabbit with a digestive tract that is still developing, with great changes taking place in the intestinal microbiota.

Digestive development and capacity depend on the diet given to the young rabbit around weaning. At this time, young rabbits have special needs that differ from those of the mother [14,27]. Diet has a strong effect on gut environment and microflora [22], because it influences the establishment of flora and fermentation parameters, as well as the development of pathogens [14].

In recent years, studies involving different ingredients/nutrients and the effect they have on the gut barrier function, rabbit performance and mortality rate caused by digestive disorders have shown that different nutritional strategies have a positive effect in limiting ERE incidence. These nutritional strategies are related to the following: (a) minimal and maximal levels of insoluble fibre (30–36% neutral detergent fibre) with specific characteristics (>3.5% acid detergent lignin) [28,29,30,31]; (b) a minimum level of 12% of inclusion of soluble fibre [32,33,34]; (c) the type and level of protein included in the diet (14–16% crude protein, 0.5–1% glutamine; preference for sunflower and animal plasma proteins over soya, pea and potato proteins) [23,35,36,37,38,39,40,41,42,43]; (d) limiting the amount of calcium in the diet [44]; and (e) the use of some prebiotics such as cellobiose combined with a low level of soluble-fibre diet [45,46].

The positive effect of soluble fibre is usually obtained using sugar-beet pulp. The combination of both soluble and insoluble fermentable fibre of sugar-beet pulp has some beneficial effects on the intestinal mucosa in the post-weaning period, such increases in (i) the ratio between the villous height and crypt depth, (ii) the number of goblet cells per villus, (iii) sucrose activity in the jejunal mucosa and digestibility of starch in the ileum, and (iv) ileal flow of mucin [43,47,48,49]. However, it has not yet been confirmed whether these effects could also be obtained with other ingredients rich in soluble/fermentable fibre.

Another strategy related to feed management that usually has positive effects on rabbit health is feed restriction after weaning [50,51,52,53]. However, all of these strategies cannot completely protect rabbits against outbreaks of ERE and might require to be combined with others such as the use of new bioactive compounds with prebiotic activity [13].

The term prebiotic is very broad and includes “any substrate that is selectively utilized by host microorganisms conferring a health benefit” [54]. Dietary prebiotics are selectively fermented ingredients that result in specific changes in the composition and/or activity of the gastrointestinal microbiota, thus conferring benefits to host health [55]. These ingredients must fulfil three criteria to be classified as prebiotics [56]: (1) they must resist gastric acidity, hydrolysis by mammalian enzymes and gastrointestinal absorption; (2) they must be fermented by the intestinal microflora; and (3) they must selectively stimulate the growth and/or activity of intestinal bacteria associated with health and wellbeing. The stimulation of these beneficial microbes enhances competition against undesirable microbes, prevents the adhesion of pathogens to the mucosa and directly stimulates the gut immune system [57]. Anyway, the potential symbiotic microbiota is not identified in rabbits, and this is an important drawback to find prebiotics that may improve intestinal health.

During the last decades, a great effort has been made to identify prebiotic compounds for routine use in the feed industry. Most of this work has been performed in the main livestock species, namely, pigs and poultry (for a review, see [24,58,59]), although great effort has also been made in the minority rabbit sector (for a review, see [57]). The most studied and well-known prebiotics are oligosaccharides (namely, mannan-oligosaccharides (MOS), fructo-oligosaccharides (FOS), a-galactooligosaccharides (GOS), cellobiose and xilo-oligosaccharides (XOS)), inulin and yeast cell products, although there are also other candidates, such as polyunsaturated fatty acids (PUFAs). Some soluble fermentable fibres and other types of dietary fibre have been also tested [24,54,57,58]. Although numerous positive effects of different types of prebiotics have been reported to reduce the incidence of diarrhoea in piglets during the post-weaning period, further research is needed to understand the influence of these compounds in gut health and consequently to enable their widespread use in animal husbandry practice [58]. This also applies to rabbit farming, and although some positive results have been observed, the lack of consistent findings precludes robust conclusions from being reached, which may be accounted for by the differences in intestinal microbiota and gut physiology among species.

## 4. Seaweed and Gut Health

Seaweed has traditionally been used in animal nutrition in some parts of the world—as fodder during periods of scarcity and also as a mineral supplement [60,61,62]. However, the current interest in the use of algae in animal nutrition arises from the urgent need to search for new bioactive substances that can improve animal health and the sustainability of animal production [63,64,65]. In this sense, seaweeds represent a very promising source of numerous beneficial substances, as they are very rich in polysaccharides with prebiotic potential [66,67,68,69].

The chemical composition and the bioactive metabolite content of seaweed/marine algae have been extensively studied, along with the variations related to species and genera, harvesting season, environmental conditions and geographical location (for a review, see [60]), and it is outside of the scope of this paper to conduct a comprehensive review of these aspects. Overall, macroalgae are classified in three groups according to their pigmentation: green seaweeds (Chlorophyta), brown seaweeds (Phaeophyta) and red seaweeds (Rhodophyta) [63,70]. Although the composition is very variable, together, these seaweeds provide numerous nutrients of great interest (in animal nutrition). The protein content varies among groups, with red algae having the highest percentage (up to 47%) and brown algae the lowest (5–15%), and it is generally of very good quality in all types due to the high content of essential amino acids [69]. Seaweeds are a rich source of minerals, as they contain high levels of potassium, sodium and calcium, as well as iron, zinc, iodine, manganese, copper, cobalt and selenium [63,71,72] and also high levels of vitamins, especially vitamins A, C and E and the B group vitamins (B1, B2 and B12) [71,73]. Seaweeds also contain large amounts of PUFAs, particularly omega-3 and omega-6, which are present in a balanced ratio [69,71,72]. They are also rich in polyphenolic compounds (such as flavonoids and tannins), which act as strong antioxidants [69,70,71,73].

The main current interest in the use of seaweeds in animal nutrition lies in their high contents of complex polysaccharides and oligosaccharides, which are not generally digested in the small intestine and are thus partially or fully fermented in the large intestine or colon, providing a rich source of dietary fibre (25–75% of DM) [61,71,73]. Brown seaweeds contain soluble fibres such as alginates, fucoidans and laminarins; green seaweeds contain ulvans, galactans, xylans and mannans; and red seaweeds are mostly composed by agars, carrageenans, xylans and porphyran [69,74,75,76,77]. Some of these polysaccharides, such as laminarins, fucoidans, alginates, galactans and ulvans, have been demonstrated to have prebiotic activity [67,71,78], among other properties (Table 1; [74,75]). These polysaccharides are fermented by and stimulate the growth of commensal bacteria and also inhibit the growth and adhesion of pathogens and improve gut architecture [61,76,77]. Improved gut health is reflected at many levels, as the consumption of algae increases the absorption of nutrients and thus growth and animal welfare [61,63]. Some of these compounds also display immunomodulatory and anti-inflammatory activities [71].

Very few studies have evaluated the inclusion of seaweed in the diets of rabbits, and in most of the studies, the main objective has been to evaluate the potential prebiotic effects on gut health. Two studies performed in Egypt [72,79] evaluated the effect of including whole algae (sun-dried) on the growth performance and gut health of growing rabbits. In both cases, the inclusion of 1% *Ulva lactuca* had positive effects on the growth performance and digestive health parameters of rabbits. On the contrary, in a study conducted in Brazil [80], in which rabbits were fed *Lithothammium* flour (up to 1% of the diet), no significant effects on animal performance or digestive health were observed, even though the highest concentration of algae (1%) led to a decrease in the length and width of the villi. Finally, studies were recently conducted in Italy with the main objective of evaluating the effects of natural extracts from plants and algae (including polysaccharides from brown seaweeds) on the reproductive performance of does [81], semen quality in bucks [82] and zootechnical performance and antioxidant effects; although no significant effects on the reproductive endpoints were observed, the supplementation of the diets with algae improved the antioxidant status and fat metabolism in the animals. In a similar study carried out in Italy, the effect of natural extracts from plants and algae on the growth performance and meat quality parameters of growing rabbits was evaluated [9,10]. The long-term supplementation of lactating does and their offspring with brown seaweed and plant polyphenols (0.3 and 0.6%) improved growth performance, lowered cholesterol content and enhanced the oxidative stability and sensory quality of the meat, leading the researchers to conclude that a low dose of brown seaweed (*Laminaria* spp.) and plant-extract supplementation (phenolic acid, hydroxycinnamic acids, tannins and flavonoids) could enhance growth performance and produced better-quality rabbit meat. Although none of these studies evaluated the gut health of rabbits, most indicated that seaweed consumption can potentially enhance growth performance and antioxidant status and produce better-quality meat.

**Figure 2:**
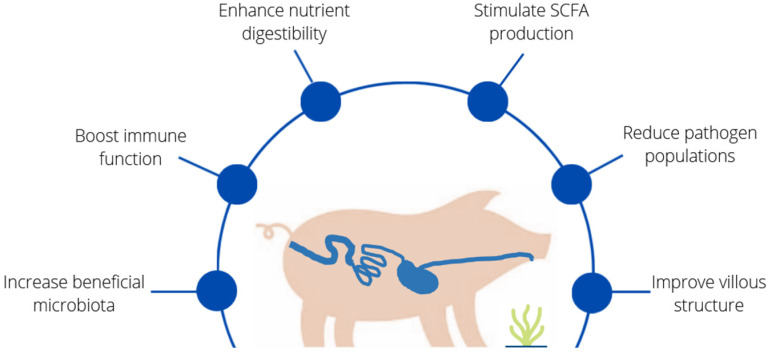
Benefits of seaweeds on piglet gut health. Increase beneficial microbiota [84,85,87,88,92,93,95], boost immune function [85,91,95], enhance nutrient digestibility [84,85,86,87,89,96], stimulate SCFA production [91,97], reduce pathogen populations [86,87,88,92,93,95,97], improve villous structure [87,97].

On the contrary, a large body of research has been carried out in piglets to evaluate the inclusion of macroalgae and algal extracts (mainly laminarin and fucoidans) in the diet in the post-weaning period. Pig production faces problems similar to those of rabbit production related to dysbiosis during the post-weaning period, which was traditionally controlled with the use of antibiotic-medicated feed and supplementation with high levels of minerals (particularly copper and zinc) [58]. The ban on (or limited use of) antibiotics has led to the need for feeding strategies in the post-weaning period that can re-establish the gut eubiosis lost at weaning, aimed at restoring the *Lactobacillus* count, promoting the growth of beneficial bacteria that boost the mucosal immune system and lowering the proliferation of pathogenic bacteria [83]. The pig farming sector already has some experience in using seaweed to improve the health and performance of piglets while avoiding the use of in-feed antibiotics (for a review, see [58,61,63]). The results of the numerous studies carried out in this field are summarized in Figure 2. Most studies have been performed with laminarin and fucoidan, which have been demonstrated to have many valuable properties. These compounds increase beneficial microbiota, enhance nutrient digestibility, improve villus structure, stimulate SCFA production, reduce pathogen populations and boost immune function, ultimately reducing post-weaning diarrhoea [61,65,66,84,85,86,87,88,89,90,91,92,93,94,95,96,97].

Given the experience with piglets, it seems reasonable to assume that rabbits could benefit from the supplementation of some algae or algae extracts enriched in polysaccharides to improve the health of the immature digestive system in the post-weaning period. This possibility deserves further study.

## 5. Seaweed and Meat Quality

Rabbit meat is a high-quality product due to its nutritive and dietetic properties [1,98]. It has a high protein content (of about 22%), characterised by high essential amino-acid levels. It is also a good source of potassium, phosphorous, selenium and B vitamins (being one of the richest sources of vitamin B12) and has a very low sodium content [2,8,98]. Moreover, the fatty acid profile of rabbit meat is considered very healthy, because the meat contains lower levels of cholesterol and saturated fatty acids than other meats and is also very rich in PUFAs, which are well balanced between the n-3 and n-6 series [99]. However, the high content of PUFAs makes this meat susceptible to oxidative deterioration and the generation of toxic compounds, which alter the sensorial properties and limit its shelf-life. Therefore, it is very important to increase the levels/balance of antioxidants in rabbit meat to guarantee its stability [99]; this can be performed by including antioxidants (preferably of natural origin) in the animal feed.

**Figure 3:**
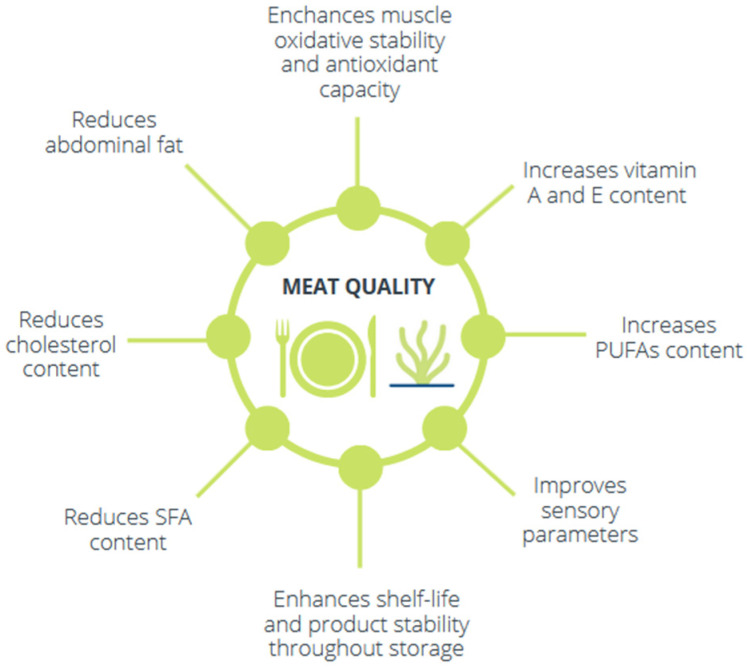
Benefits of seaweeds on meat quality.

Seaweeds are a rich source of antioxidants such as polyphenols and vitamins [60,71,100,101], and numerous studies have demonstrated their usefulness in improving muscle oxidative stability and antioxidant capacity in the main livestock species (Figure 3). The inclusion of laminarin and fucoidan derived from *Laminaria digitata* in piglet diets reduces lipid oxidation in the fresh meat [11,102] and also improves its antioxidant capacity [102]. Another study [103] has observed an effect on meat colour that may be related to an increase in antioxidant compounds [101]. This effect has also been observed in ruminants [104,105] and in broiler chickens [106].

It has also been observed that some seaweeds *(L. digitata, L. japonica, A. nodosum*) enhance the fatty acid profile of the meat by increasing the PUFA content and reducing the levels of saturated fatty acids and cholesterol, as observed in piglets [12], ducks [107] and cattle [108]. A reduction in abdominal fat in broiler chickens has also been observed when fed a diet including *Ulva lactuca* [109]. The inclusion of laminarin and fucoidan in piglet diets also enhanced the visual sensory descriptors of the meat [12] and reduced bacterial counts during storage [102]. Finally, the high iodine content of some brown algae, easily transferred to animal tissues, has been proposed as a potential means of mitigating iodine deficiency in humans, with health benefits regarding the prevention of thyroid dysfunctions [85].

Information about the capacity of seaweeds to improve meat quality in rabbits is scarce. The short- and long-term inclusion of *Laminaria* spp. (0.3 and 0.6%) in rabbit diets was recently studied [9,10]. In a 42-day-long trial in growing rabbits, the vitamin A and E contents of muscle were improved, enhancing nutritional quality and oxidative stability; the sensory parameters were also enhanced. When lactating does were given the algal supplement, their offspring showed a reduction in cholesterol content and an increase in α-tocopherol and retinol contents, while the sensory quality of the meat was also improved.

Altogether, the available information indicates that the inclusion of macroalgae in the diet of rabbits could further improve the intrinsic properties of the meat and enhance its stability. This approach could be used as a marketing tool to help to generate a niche market for rabbit meat as a healthy food [1].

## 6. Seaweed and Sustainable Animal Farming

Seaweed farming is increasing worldwide as part of the development of a sustainable economy [110]. China and Indonesia, where harvesting or collecting algae from the natural environment is an ancient practice, are the major seaweed-producing countries, contributing 86.6% of global seaweed production [60,111]. Within Europe, France is also a top producer of algae [111], and seaweed farming is currently expanding in Mediterranean countries [112]. Seaweeds are used for multiple purposes, directly as animal feed and to produce biofertilizers. They can also be refined to extract some compounds of interest for the pharmaceutical, cosmetic and food industries, and by-products are used to produce biofuels or biofertilizers [112,113,114,115].

The use of macroalgae as an alternative to the use of antibiotics in animal production has many environmental benefits, beginning with the direct reduction in the discharge of pharmaceutical residues in the environment [116]. The global consumption (228 countries) of antimicrobials in food animal production was estimated to be 63,151 (±1560) tonnes in 2010 and is expected to rise by 67% in 2030, almost doubling in BRICS countries [117]. Between 40 and 90% (depending on the class of drugs) of the antibiotic dose administered is excreted as parent compounds in the active form in the faeces and urine, eventually reaching the environment and contaminating soils, water and plants [118]. Once in the environment, antibiotic residues can have negative effects on biota at different trophic levels and on human health via the consumption of contaminated food and water, also contributing to increasing the resistant bacterial population and maintaining selective pressure that leads to the development and/or dissemination of resistance in different environmental compartments [119,120].

The inclusion of seaweed as an ingredient in animal feed provides essential amino acids, PUFAs, vitamins and minerals, antioxidants and soluble fibre, which together can improve animal health and contribute to low mortality rates and a reduction in antibiotic use [58,60]. The minimization of mortality rates would considerably reduce the environmental impact of rabbit farming by increasing its efficiency [8,121]. To achieve this goal, the development of diets that are well adapted to the post-weaning period together with improved farm management and hygienic conditions are crucial [8,14].

It is well known that the greatest environmental impact of monogastric farming is associated with feed production, especially commercial protein feeds such as soybean meal, which are associated with deforestation and emissions derived from transport [8,122]. Reduced protein intake and the substitution of part of the protein with other sources with lower environmental impact, such as macroalgae, would, therefore, contribute to mitigating damage to the environment and pollution due to nitrogen excretion [8,123,124]. Moreover, in the context of population growth and limited sources, high rates of production of marine seaweeds could be achieved without the need for fresh water, arable land or fertilisation; seaweeds, therefore, represent an interesting source of ingredients and bioactive compounds for human and animal nutrition [61,97,100,123,125].

Within sustainable farming systems, seaweed cultivation is of interest because of the ecosystem services that algae provide [110]. Macroalgae possess bioremediation properties, as they are capable of minimising eutrophication by removing excess dissolved nutrients such as C, N and P [126,127]. They also remove heavy metals from water [128] and act as a potential carbon sink that contributes to mitigating ocean acidification and climate change [100,114]. In this sense, seaweed cultivation has been proposed as a beneficial co-culture practice in aquaculture, in a new system called IMTA (integrated multi-trophic aquaculture), in order to reduce the environmental impact of fish and mussel production [127]. Intensive mariculture produces large quantities of organic and inorganic pollutants that cause environmental deterioration [126]. The IMTA system combines the cultivation of various species from different trophic levels and complementary ecosystem functions (fed species (fish/shrimp), filtering species (mussels/oysters/other molluscs) and extractive species (seaweeds)), so that the waste and nutrients derived from one culture can be reused and used for other species [127,129]. As a result, the whole system is less harmful to the environment [126,129], and total production is higher than that of monoculture systems, reaching higher biomass yield [130] and better-quality products [127]. In Europe, there is already some experience with the IMTA system, in some countries such as France, Germany, Norway, the UK, Ireland, Portugal and Spain [129,131], although it is not yet as widespread as in Asia [132]. The commercial value of the seaweed used in these systems is a key point regarding the potential profitability of IMTA [129,133]. The inclusion of algae in livestock feed could increase the commercial value of algae, especially in some European countries where livestock production is economically important, so that both sectors could reinforce each other [129]. The fact that there is already a market for algae in the main rabbit-producing countries means that its use in animal feed as a local-produced alternative to antibiotics could be used as a marketing strategy for consumers who are increasingly concerned about issues of environmental sustainability.

**Figure 4:**
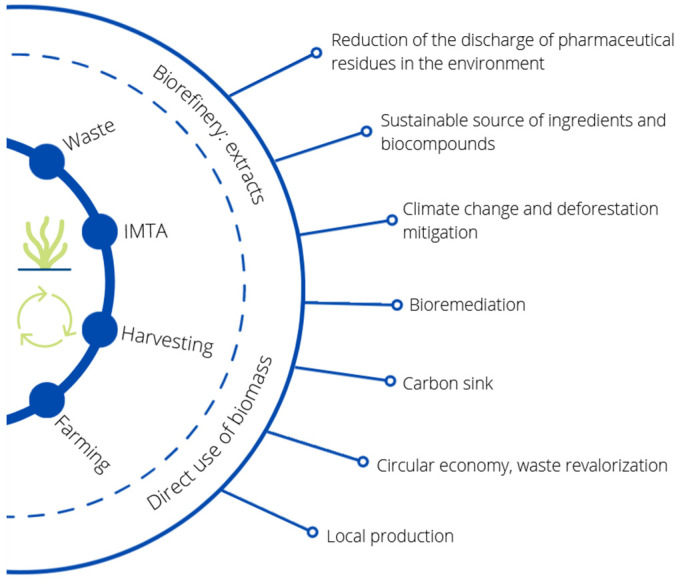
Environmental benefits of revalorizing seaweeds for animal feed.

Furthermore, some seaweeds such as *Ulva* spp. grow uncontrollably on the coast and have some negative environmental impacts, leading to coastal degradation and problems for the fishing industry and tourism. Seaweed is removed regularly, thereby generating tons of marine macroalgal waste every year [62,69]. In addition, the seaweed industry also generates large amounts of waste during the transformation process. This waste could be revalorized for inclusion in animal feed, among other uses, providing benefits both to animal health and the economic viability of industries. This approach would create a circular economy model that would be beneficial in environmental, economic, social and animal welfare terms (Figure 4) [62,129].

## 7. Conclusions and Future Trends

The rabbit meat industry is unquestionably facing critical challenges that require a holistic solution. On the one hand, the ban on the prophylactic use of antibiotics in animal nutrition has led to changes in the rabbit diet (to improve gut health) and is one of the key points regarding the economically viability of rabbit farming (along with farm husbandry and hygiene conditions). On the other hand, a niche market that offers new formats of meat adapted to modern forms of consumption must be created to compete with other types of meat on the market. Thus, a distinctive meat product that can attract consumers who are increasingly more aware of healthy and sustainable products is required.

This review paper highlights the potential value of macroalgae in the rabbit farming sector. In line with sustainable agriculture programmes, and particularly with the European Green Deal Plan, the use of seaweed in rabbit nutrition may improve animal health according to the One Health approach, whereby consumers and the environment could receive tangible benefits. The rabbit sector also needs a powerful marketing campaign to attract consumers concerned about the environmental sustainability of animal nutrition, and producing higher-quality meat can attract new consumers.

Although algae have been extensively analysed regarding their contents of biologically active compounds, the potential benefits in animal feed, and particularly in rabbit feed, requires further research. Demonstrating the prebiotic effects of algal components on gut health and also identifying the compounds directly responsible for the antimicrobial, antiviral, antioxidative and anti-inflammatory properties of algae remain incipient lines of research. Focusing on the use of algae to reinforce intestinal health, once the use of antibiotics is limited, through a viable and sustainable approach is a strategy that represents progress in solving the major emerging problems related to antibiotic resistance.

## Figures and Tables

**Table 1:** Main polysaccharides of interest in macroalgae.

Polysaccharide	Chemical Structure	Biological Properties
Ulvan	L-rhamnose, D-xylose, D-glucose and D-glucuronic acid.	Antioxidant, anticoagulant, anti-inflammatory, immune-stimulatory, antibacterial, antihyperlipidemic.
Fucoidan	1,3-α-fucopyranoside backbone with branching of α-1,2-fucopyranoside	Antioxidant, anticoagulant, anti-inflammatory, antiviral, immune-modulatory.
Laminarin	1,3-β-D-glucan with β-1,6-linkages.	Antioxidant, anticoagulant, anti-inflammatory, antiviral, immune-modulatory, prebiotic.
Alginate	1,4-β-D-mannuronic acid and α-L-guluronic acid residues.	Antioxidant, anti-inflammatory, antitumor.

## Data Availability

Not applicable.
